# Effects of Fasting on Metabolic Hormones and Functions: A Narrative Review

**DOI:** 10.14789/jmj.JMJ24-0012-R

**Published:** 2024-10-15

**Authors:** JULIUS FINK, MASAMI TANAKA, SHIGEO HORIE

**Affiliations:** 1 Department of Urology, Juntendo University, Graduate School of Medicine, Tokyo, Japan; 1 Department of Urology, Juntendo University, Graduate School of Medicine, Tokyo, Japan; 2 School of Medicine, Tokyo Women’s Medical University Adachi Medical Center, Tokyo, Japan; 2 School of Medicine, Tokyo Women’s Medical University Adachi Medical Center, Tokyo, Japan; 3 Department of Urology, Advanced Informatics of Genetic Diseases, Digital Therapeutics Juntendo University, Graduate School of Medicine, Tokyo, Japan; 3 Department of Urology, Advanced Informatics of Genetic Diseases, Digital Therapeutics Juntendo University, Graduate School of Medicine, Tokyo, Japan

**Keywords:** fasting, testosterone, metabolism, diet, hormones

## Abstract

The occurrence of the metabolic syndrome and its related diseases such as diabetes are steadily rising in our modern society. Modern food choices and the more sedentary lifestyles largely contribute to this shift in our society’s health. Fasting has been practiced for religious purposes all over the world long time before science showed the benefits of it. The effects of fasting on glucose and fat metabolism are of great interest. Fasting triggers a cascade of changes in the hormonal, microbiome and enzymatic environments, leading to shifted glucose and fat metabolisms. Fasting-induced metabolic function changes are affected by several factors such as sex hormones, lipid-released hormones, growth hormone, insulin, and the gut microbiome, leading to lipolysis and the release of FFA into the bloodstream.

The purpose of this review is to summarize the newest research results on the specific pathways fasting triggers to improve metabolic functions and understand the potential applications of fasting as prevention/treatment of several metabolic conditions.

## Introduction

Fasting can lead to major changes in glucose and lipid control, however, the effects of fasting are not completely understood to this day. Recent diet trends such as intermittent fasting, and traditional religion-based fasting protocols have been intensively investigated during the last decade. Recent studies showed that eating in a 6-hour period and fasting for 18 hours might modify energy sources from glucose-based to ketone-based energy, leading to improved stress resistance, longevity, and delayed onset of diseases such as cancer and obesity^1)^. The cellular and molecular mechanisms involved depend on the activation of adaptive cellular stress response signaling pathways improving mitochondrial health, DNA repair, autophagy, stem cell-based regeneration, and chronic metabolic effects^2)^. A recent study found that a 30-day period of fasting from dawn to sunset for >14 hours without caloric restriction led to upregulated key regulatory proteins of glucose and lipid metabolism, circadian clock, DNA repair, cytoskeleton remodeling, immune system, and cognitive function, and resulted in a serum proteome protective against cancer, metabolic syndrome, inflammation, Alzheimer’s disease, and several neuropsychiatric disorders^3)^. Fasting also affects several hormones involved in glucose and fat metabolism. Fat-derived hormones such as leptin and adiponectin and sex hormones such as testosterone and estrogen can also greatly affect glucose and fat metabolism. Even growth hormone (GH), a major regulator of lipolysis, is affected by fasting. The gut microbiota also seems to play an important role in the effects of fasting on glucose and fat metabolism. Several studies have been conducted on the effects of fasting, however it is not clear how each of the proposed mechanisms act together to induce the health benefits of fasting. Moreover, research directly comparing several fasting durations is also lacking. This review will look at each potential pathway involved in the effects of fasting and different fasting durations on metabolic functions and help to understand the underlying mechanisms.

## Fasting

Fasting is widely used to describe a caloric or time restriction. However, there are many different ways fasting can be conducted. Some studies look at strict fasting, meaning no food at all, whereas others look at intermittent fasting, meaning time restriction for food intake during the day, or caloric restrictions. Furthermore, the exact modalities of intermittent fasting are not universally specified. Several different forms of intermittent fasting have been utilized in past research, ranging from time- restricted fasting without calorie restriction, such as the Ramadan fasting protocol, the 16/8 protocol (16 hours of fasting and 8 hours of normal eating), the 5 : 2 diet (eating normally for 5 days of the week and restricting caloric intake to 500-600 on 2 days, alternate-day fasting and many more versions^4)^. Given the large discrepancies in the fasting methods, it is difficult to compare several studies, especially in a meta-analysis. Therefore, we chose to conduct a narrative review. Table 1 summarizes some of the major studies conducted on fasting and glucose and fat metabolism in humans. The major outcomes are glucose and lipid metabolism and hormonal changes.

**Table 1:** Major studies conducted on fasting and glucose and fat metabolism in humans

Authors	Year	Participants	Method of fasting	Outcomes
Liu et al.	2021	76 women	24 hours on 3 non consecutive days per week for 8 weeks	1) Increased antioxidant activity 2) Increased insulin resistance
Arnason et al.	2017	n=?, type 2 diabetes patients	18-20 hours of daily fasting for 2 weeks	1) Weight decrease 2) Morning glucose decrease
Corley et al.	2018	41 patients with type 2 diabetes	2 consecutive days of very-low-calorie diet vs. 2 non-consecutive days of very-low-calorie diet	No difference between interventions 1) Improved HbA1c 2) Improved fasting glucose
Kunduraci et al.	2020	70 patients with metabolic syndrome	Intermittent Energy Restriction (16 hours, 25%) vs. Continuous Energy Restriction (25%) for 12 weeks	Blood pressure, total cholesterol, triglyceride, low-density lipoprotein (LDL), fasting glucose, and insulin at the 12th week decreased in both groups
Harder-Lauridsen et al.	2017	10 healthy lean men	14 hours daytime (Ramadan) for 28 days	Minor effect on body mass index and without any effects on body composition, glucose metabolism, and cognitive function
Mindikoglu et al.	2020	14 healthy individuals	14 hours dayytime for 30 dyas	anticancer serum proteomic signature, upregulated key regulatory proteins of glucose and lipid metabolism, circadian clock, DNA repair, cytoskeleton remodeling, immune system, and cognitive function
Moro et al.	2016	34 resistance trained men	16 hours fast for 8 weeks vs. control	Testosterone and insulin-like growth factor 1 decreased significantly in the fasting group, with no changes in control. Adiponectin increased in fasting group and total leptin decreased
Mesbahzadeh et al.	2005	52 male students	> or = 12 hours during Ramadan (28 days)	Testosterone decreased after 20 and 28 days. FSH increased after 20 days. LH did not change.
Shulte et al.	2014	13 obese men	Very low calorie diet (800 kcal/d) for 12 weeks	Serum testosteorne increased via 2 distinct mechanisms 1) Improvement of testicular function 2) Reduced conversion of testosterone to β-estradiol by aromatase activity of the adipose tissue.
Ho et al.	1988	6 normal men	5 days fast	GH pulse frequency, amplitude and concentration increased Glucose decreased FFA increased
Nørrelund et al.	2001	8 normal subjects	1) in the basal postabsorptive state (basal), 2) after 40 h of fasting (fast), 3) after 40 h of fasting with somatostatin suppression of GH (fast-GH) 4) after 40 h of fasting with suppression of GH and exogenous GH replacement (fast+GH)	IGF-I and free IGF-I decreased 35 and 70%, respectively, during fasting without GH Muscle-protein breakdown was increased among participants who fasted without GH
Gabel et al.	2019	43 insulin resistant individuals	12-month study that compared ADF (25% energy needs on “fast days”; 125% energy needs on alternating "feast days") with CR (75% energy needs every day) and a control group regimen.	Weight loss was not different between ADF (-8% ± 2%) and CR (-6% ± 1%). Fat mass and BMI decreased similarly from ADF and CR. ADF produced greater decreases in fasting insulin (-52% ± 9%) and insulin resistance (-53% ± 9%) compared with CR (-14% ± 9%; -17% ± 11%) and the control regimen after 12 months.
Furmli et al.	2018	3 men with type 2 diabetes	24-hour fasts three times per week over a period of several months	Complete discontinuation of insulin in all patients. The minimum number of days to discontinuation of insulin was 5 and the maximum was 18. There was a general reduction of haemoglobin A1C (HbA1C) levels for all patients during the course of the fast.
Vendelbo et al.	2012	8 healthy subjects	72-h fast vs. 10-h overnight fast.	Skeletal muscle insulin sensitivity was reduced profoundly in response to a 72-h fast and substrate oxidation shifted to predominantly lipid oxidation. Both lipid and glycogen accumulated in skeletal muscle. Intracellular insulin signaling to glucose transport was impaired by regulation of phosphorylation at specific sites on AS160 but not TBC1D1, both key regulators of glucose uptake. In contrast, fasting did not impact phosphorylation of AMPK or insulin regulation of Akt, both of which are established upstream kinases of AS160. These findings show that insulin resistance in muscles from healthy individuals is associated with suppression of site-specific phosphorylation of AS160, without Akt or AMPK being affected. This impairment of AS160 phosphorylation, in combination with glycogen accumulation and increased intramuscular lipid content, may provide the underlying mechanisms for resistance to insulin in skeletal muscle after a prolonged fast.

## Fasting and lipid metabolism

Fasting triggers shifts in fuel sources. Free-fatty acid (FFA) mobilization, fatty-acid oxidation (FAO), and ketogenesis progressively increase, leading to the conservation of glucose.

It has been hypothesized that increased FFA delivery can lead to excessive and incomplete FAO, potentially triggering skeletal muscle insulin resistance. A major factor seems to be lipid-induced mitochondrial stress, which is the main site of endogenous reactive oxygen species production.

Obese individuals tend to have increased lipid deposition in skeletal muscle^5)^. Interestingly, acute, prolonged fasts, can lead to the development of insulin resistance^6)^. However, the onset of insulin resistance in response to fasting might be a protective reaction to maintain glucose levels without hyperglycemia and hyperinsulinemia^7)^. One recent study investigating the effects of 24 hours of intermittent fasting showed an increase in non-esterified fatty acids and *β*-hydroxybutyrate^8)^. This supports the hypothesis that intermittent fasting increases the utilization of stored body fat.

## Fasting and glucose metabolism

Insulin resistance is a driving factor for impaired glucose metabolism. The loss of body fat and the maintenance of lean mass is crucial for healthy glucose control. Intermittent fasting often leads to reductions in body mass, therefore improving glucose control. However, besides the loss of body mass, several genes controlling glucose metabolism seem to be affected during fasting.

One time-restricted intermittent fasting (average 16.8 hours of fast/day) for 2 consecutive weeks showed a 6.1% reduction in fasting glucose and a 6.1% reduction in HOMA-IR^9)^. Another intermittent fasting approach, very low caloric intake (approximately 500-600 kcal) 2 days per week, on 2 consecutive or nonconsecutive days has shown significant reductions in glucose and HbA1c levels in T2DM patients^10)^. Interestingly, another study found that a continuous energy restriction of 25% leads to similar effects on glucose metabolism biomarkers (glucose, HOMA-IR, HbA1c) as a 16- hour intermittent fasting energy restriction diet after 12 weeks^11)^. One study looking at the effects of Ramadan (fasting for 14 hours for 28 days) on blood glucose showed no significant improvements on blood glucose markers^12)^.

Genetical adaptations to fasting have also been investigated in one recent study.

This study looked at the proteome after 30 days of >14 hours fasting, and showed significant upregulation of several signature genes involved in cytoskeleton remodeling, glucose, and lipid metabolism. Tropomyosins are essential proteins composing actin cytoskeleton in non-muscle cells increased after the fasting period. It has been observed that impaired function of the actin cytoskeleton can lead to down-regulated exocytosis of glucose transporter (GLUT4), ultimately triggering insulin resistance^3)^. Moreover, TPM3 gene encodes for tropo-myosin 3.1, a key player in remodeling insulin-induced actin cytoskeleton, leading to improved insulin sensitivity. TPM4 gene encodes for a protein binding to cytoskeletal actin, improving insulin responsiveness. Fasting might also improve insulin resistance via upregulation of PLIN4 (Perilipin 4) expression, triggering similar effects as PPAR-*γ* (Peroxisome proliferator-activated receptor gamma) activators (e.g., pioglitazone). CFL1 (Cofilin 1), thought to play a critical role in insulin-induced GLUT4 translocation and glucose uptake, has also been shown to increase after 30 days of fasting^3)^. PKM (Pyruvate kinase isozymes), encoding the pyruvate kinase enzyme, and being a main regulator of glycolysis has also been shown to increase after fasting^3)^. The upregulation of TPM3, TPM4, PLIN4, CFL1, and PKM expression indicate that intermittent fasting might be effective in the prevention and treatment of the metabolic syndrome^3)^. Serum proteomics indicate protective effects of fasting on metabolic functions, which might not have been detected with serum biomarkers.

One recent study showed that postprandial carbohydrate oxidation was significantly decreased after a 72- vs. 13-h fast (P < 0.0001), while fat oxidation was significantly upregulated (P < 0.0001)^13)^. 72 h of fasting induced greater glucose and insulin fluctuations in response to food intake were as compared with the 13-h fast (P < 0.001). Interestingly, the 72 h fast group had a significant reduction in glucose tolerance^13)^.

## Fasting and protein metabolism

Amino acids are the building blocks for proteins. Fasting has been shown to affect not only amino acid content but also amino acid composition. One study on starvation showed a transient increase in plasma valine, leucine, isoleucine, methionine, and α-aminobutyrate during the first week, while a delayed, progressive upregulation in glycine, threonine, and serine was observed after 5 days^14)^. Previous research showed that after 3 hours of fasting, plasma lactic acid, total amino acids, and the total amount of essential amino acids significantly decreased^15)^. However, glycerin, free fatty acids, *β*-hydroxybutyric acid, and acetoacetic acid increased, and arginine, alanine, serine, threonine, and aspartic acid and proline decreased. Some amino acids remained at similar levels during fasting. Interestingly, lysine, leucine, isoleucine, and taurine decreased up to 6 hours of fasting, before recovering after 12 hours of fasting. Also, essential amino acids decreased more as compared to nonessential amino acids^15)^. It is well understood that essential amino acids are crucial for muscle growth and repair, while non-essential amino acids regulate immune function and energy production. Non- essential amino acids also have the ability to be converted into glucose, a major source of energy. This could be a mechanism of the body to prioritize energy, rather than building muscle in times of fasting. One study showed that regulation of amino acid catabolism genes during fasting is regulated by glucagon (via CREB) and corticosterone (via GR), while glucose production in hepatocytes increases, suggesting that glucagon alone is not enough to fully induce gluconeogenesis^16)^.

## Intermittent fasting and fat derived hormones

Leptin signals the brain to regulate appetite via LepR-receptor binding in the hypothalamus, leading to the activation of a complex neural circuit including anorexigenic (i.e. appetite-diminishing) and orexigenic (i.e. appetite-stimulating) neuropeptides. Leptin also activates the mesolimbic dopamine system, regulating motivation for and reward of feeding, and the nucleus of the solitary tract of the brainstem activating satiety^17)^. Adiponectin is a hormone and an adipokine protein regulating numerous metabolic processes, including insulin-sensitizing and anti-inflammatory properties. In healthy individuals, leptin inhibits the biosynthesis and secretion of insulin from pancreatic *β*-cells. On the other hand, insulin activates the secretion of leptin from adipose tissues. Leptin also triggers hepatic gluconeogenesis, glucose uptake in skeletal and cardiac muscle^18)^. In leptin-resistant individuals, poor leptin signaling in the hypothalamus can lead to hyperglycemia and hyperinsulinemia^19)^. One meta-analysis investigating the effects of Ramadan fasting on leptin and adiponectin showed that leptin decreases while adiponectin remains unaffected after Ramadan fasting^20)^. Leptin secretion is mainly activated via insulin-induced alterations in adipocyte metabolism. Alterations of insulin concentrations triggered by lower energy intake during Ramadan may therefore lead to shifts in leptin concentrations. Moreover, meal timing alternates the diurnal rhythm of leptin by alterations in insulin secretion^20)^. Another meta-analysis showed that fasting and energy-restricted diets lead to drops in leptin^21)^. With regard to adiponectin, increases may also be achieved when energy intake is ≤50% of normal maintenance^21)^. Adiponectin is anti-inflammatory and anti-atherogenic and adiponectin may be negatively correlated with visceral adiposity and insulin resistance^22)^. Also, the ratio of adiponectin/leptin can be a marker of insulin resistance, more so than adiponectin or leptin alone^23)^. The adiponectin/leptin ratio diminishes with the severity of metabolic syndrome, meaning that this ratio can be utilized as a marker for metabolic syndrome^24)^.

Taken together, these findings indicate that fasting can improve glucose metabolism via a decrease in leptin and an increase in adiponectin, depending on the level of energy reduction.

## Fasting and testosterone

In men, testosterone is a key regulator of lipid and glucose metabolism. One recent study matching the daily caloric intake and macronutrient distribution found that a diet including 16 hours of daily fasting for 8 weeks leads to decreased levels of serum testosterone in men^25)^. Interestingly, the intermittent fasting group showed increases in adiponectin which were not observed in the normal diet group.

One study looking at the effects of Ramadan fasting on serum testosterone levels showed that daily fasting ≥ 12 hours leads to decreases in testosterone from 7.17 to 6.59 after 10 days, 5.68 after 20 days (P < 0.05), and 5.92 ng/mL after 28 days (P < 0.05) of fasting^26)^.

Straight fasting for 3 days also showed a reduction of ~35% in serum total testosterone levels in healthy young men^27)^. However, to our knowledge, the current body of literature does not provide information about the effects of prolonged fasting >3 days on testosterone.

On the other hand, in obese individuals, caloric restrictions and fasting could lead to improved serum testosterone levels via 2 pathways: First, the improvement of testicular function and second, the downregulation of aromatase activity due to decrease of adipose tissue leads to a reduced conversion rate of testosterone into estrogen^28)^. The LH/T ratio, considered a marker for testicular function, significantly increased after 3 months of a very low caloric diet (800 kcal/day), pointing to an improvement in testicular function^28)^. Therefore, the effects of fasting and testosterone probably depend on the modalities of fasting and the initial body composition of the individual.

## Fasting and growth hormone

Growth hormone upregulates glucose release via gluconeogenesis and glycogenolysis from the liver and kidney, while it suppresses glucose uptake in adipose tissue. Also, GH inhibits the expression of glucose transporter 1 (GLUT1) and GLUT4 in adipocyte plasma membranes. On the other hand, GH stimulates lipolysis through upregulation of hormone-sensitive lipase, leading to free fatty acid (FFA) flux from adipose tissue into the circulation, possibly triggering insulin resistance via downregulation of insulin receptor substrate-1 (IRS-1) activity and the following impaired PI3K activation in the skeletal muscle and liver. GH enhances cellular uptake of FFA in skeletal muscle via upregulation of lipoprotein lipase expression^29)^. Interestingly, hyperinsulinemia following GH administration could be related to beta-cell compensation for insulin resistance or GH could directly upregulate beta-cell proliferation and glucose-stimulated insulin secretion^30)^.

GH has been shown to increase after a period of fasting. GH is known to upregulate hepatic glucose output, leading to positive nitrogen balance, and lipolysis^31)^. Thus, GH might be a key factor to maintain protein during fasting. Interestingly, the effects of GH on lipolysis seem to be enhanced in the fasting state as compared to the fed state. On the other hand, fasting leads to decreased insulin sensitivity and IGF-I bioactivity in response to GH administration during fasting^32)^. In other words, GH inhibits insulin’s action on skeletal muscle, liver, and adipose tissue, leading to upregulated glucose release from the skeletal muscle and liver, while glucose uptake from adipose tissue decreases^29)^.

One study showed the protein-conservative effects of GH during fasting. Participants were divided into 4 groups, normal state, 40 hours of fasting, 40 hours of fasting + somatostatin (GH inhibitor), 40 hours of fasting + somatostatin + exogenous GH replacement^33)^. As compared with the exogenous GH administration group, IGF-I decreased 35 % during fasting without GH. Urinary urea excretion and serum urea levels raised for the fasting group without GH (urea excretion: basal 392 ± 44, fast 440 ± 32, fast-GH 609 ± 76, and fast+GH 408 ± 36 mmol/ 24 h, P < 0.05; serum urea: basal 4.6 ± 0.1, fast 6.2 ± 0.1, fast-GH 7.0 ± 0.2, and fast+GH 4.3 ± 0.2 mmol/l, P < 0.01). The muscle-protein breakdown was upregulated in the fasted without GH group (phenylalanine rate of appearance: basal 17 ± 4, fast 26 ± 9, fast-GH 33 ± 7, fast+GH 25 ± 6 nmol/min, P < 0.05)^33)^. These results indicate a protective effect of GH for protein during fasting.

Interestingly, the mechanism by which fasting triggers GH increases seems to be regulated by ghrelin. One study showed that fasting induces an acute and distinct diurnal rhythm in systemic ghrelin levels followed by similar changes in serum GH levels^34)^. Ghrelin is mainly released in the stomach, therefore the stomach might affect the anterior pituitary^35)^.

In animal studies, it has been shown that intermittent fasting does not improve insulin sensitivity and maximal longevity of male GHR-KO (growth hormone receptor-ko) mice, even though these improve in non-GHR-KO males from the same strain^36)^.

## Fasting and insulin

Insulin is the key regulator of glucose metabolism. Maintaining healthy insulin sensitivity is crucial to prevent the onset of metabolic diseases. The composition and timing of food intake is very important to manage insulin release. It is well understood that carbohydrates trigger the largest spikes in insulin. However, fasting is well known to increase insulin sensitivity. One recent study compared the effects of a caloric restriction (75% energy needs every day) with alternate-day fasting (25% energy needs on “fast days”; 125% energy needs on alternating “feast days”) and a control group in obese insulin-resistant patients. Both the alternate-day fasting group (−8% ± 2%) and the caloric restriction group (−6% ± 1%) decreased body weight by month 12, as compared to controls (P < 0.0001), however, there was no significant difference among the 2 intervention groups. On the other hand, alternate-day fasting led to more pronounced decreases in fasting insulin (−52% ± 9%) and insulin resistance (HOMA-IR) (−53% ± 9%) as compared with the caloric restriction (−14% ± 9%; −17% ± 11%) and the control group after 12 months^37)^. One recent study even showed that intermittent fasting (24-hour fast 3 times/week) can help reverse type 2 diabetes and minimize the use of medications in patients with type 2 diabetes^38)^. These results show that therapeutic fasting can potentially reverse insulin resistance^38)^. On a side note, the use of Sodium Glucose Co-Transporter-2 Inhibitors (SGLT2i) has also been shown to mimic therapeutic carbohydrate restriction. Several studies investigating the effects of therapeutic carbohydrate restriction or ketogenic nutrition have demonstrated that this method can improve glycemic control, and ultimately lead to the remission of type 2 diabetes. Indeed, SGLT2i 1 glucose from the body, leading to similar effects as seen with intermittent fasting or calorie restriction in the form of very low energy diets^39)^.

Interestingly, acute fasting for a longer period (72 hours) has been shown to decrease insulin sensitivity^40)^. Indeed, during fasting, human skeletal muscle relies on lipid oxidation, potentially triggering the development of insulin resistance and impaired glucose uptake. 60-h fasting led to an approximately twofold increase in skeletal muscle lipid content and a 10% increase in glycogen, whereas 12-h fasting did not show any changes. Not only glycogen but lipid accumulation in skeletal muscle is also correlated with the onset of insulin resistance. Glycogen synthesis is downregulated by increases of glycogen in the muscle, whereas nonoxidative glucose disposal is downregulated during fasting. After 72 hours of fasting, the enzyme glycogen synthase (GS), controlling glycogen synthesis, protein expression did not change, but GS gene expression decreased by 33%. In accordance with the GS activity markers, GS phosphorylation, which inactivates the enzyme, was significantly upregulated during fasting^40)^.

These findings indicate that fasting can have positive or negative effects on insulin sensitivity, depending on the modality of fasting. Intermittent fasting seems to improve insulin sensitivity, whereas fasting for several days seems to decrease it.

## Fasting and sirtuin

Sirtuins are protein enzymes which control several cellular functions, such as aging, inflammation, detoxification, stress resistance, fat and glucose metabolism, circadian rhythms, and mitochondrial biogenesis. Seven different sirtuins (SIRT-1 through SIRT-7) regulate brain and nerves, metabolism, heart and blood vessels, and immune system.

Recent studies showed that fasting may protect normal cells and mice from metabolic conditions, and even downregulate the occurrence of carcinogenesis. Sirtuins, specially SIRT1 and SIRT3, seem to be upregulated by fasting and improve insulin response, antioxidant defense, and glycolysis^41)^.

SIRT-1 has been extensively studied for longevity, since it is up-regulated by calorie restriction, ultimately slowing the aging process^42, 43^^)^. During short term fasting, the CRTC2 activity is downregulated by SIRT1, leading to lower gluconeogenesis in the liver. During long term fasting, SIRT1 triggers PGC-1α, leading to downregulated adiposity and lipogenesis and to upregulated fatty acid oxidation^44)^.

SIRT-2 appears to be related to maintaining a healthy weight via glucose regulation^45)^.

SIRT-3 exerts potent antioxidant activity^46)^.

SIRT-4 also regulates insulin sensitivity^47)^.

SIRT-5 seems to regulate liver detoxification via conversion of ammonia to urea for excretion from the body^48)^.

SIRT-6 seems to be crucial for longevity and glucose control^49)^.

SIRT-7 seems to antagonize human stem cell aging via heterochromatin stabilization^50)^.

SIRT1, SIRT6 and SIRT7 are nuclear sirtuins, controlling multiple important transcription factors of metabolic pathways. SIRT2 is mainly found in cytoplasm and can control cellular mitosis, preventing genomic instability. SIRT3, SIRT4 and SIRT5 are found in the mitochondria^41)^. Sirtuins need NAD+ as a cofactor to deacetylate target proteins and change their function. Therefore, it has been hypothesized that sirtuins are at the key of cellular energy metabolic regulation. In a nutrient-abundant environment, glycolysis is the primary source to produce energy and induce increased NADH levels as well as decreased NAD+ levels, leading to an inhibition of the enzymatic activity of sirtuins. On the other hand, in an environment poor in nutrients such as fasting, this might increase NAD+ levels in the nucleus and cytoplasm, leading to increased enzymatic activity of sirtuins^41)^. Interestingly, testosterone has been shown to upregulate SIRT1 expression, while high glucose exposure leads to downregulation of SIRT1^51)^. High glucose can have inhibitory effects.

## Gut microbiome and fasting

Previous studies have shown that intermittent fasting might enhance the composition and diversity of the gut microflora. Fasting seems to reduce gut permeability, potentially preventing conditions commonly seen in obese populations such as systemic inflammation which are typically elevated in obesity^52)^. Reductions in nutrient load can restructure the gut microbiome by triggering the production of health-promoting bacteria and metabolites, potentially leading to improvements in hormones such as testosterone^52)^.

Changes in the gut microbiota related to metabolic diseases differ in men and women. Estrogen and testosterone seem to influence the sexual dimorphism of the gut microbiota and the development of metabolic diseases. Recent research suggests that the correlation between the gut microbiota and its host is crucial for the onset of metabolic diseases^53)^. The protection of the intestinal mucosa by the gut microbiota is important for the gut barrier^54)^, which regulates the interaction of microorganisms with the bloodstream, affecting inflammation^55)^. The microbiota not only influences the gut, but also the central nervous system via the gut-brain axis, and the liver to regulate nutrient metabolism, via the gut-liver axis^56)^.

## Fasting, testosterone and decrease in body fat

**Figure 1:**
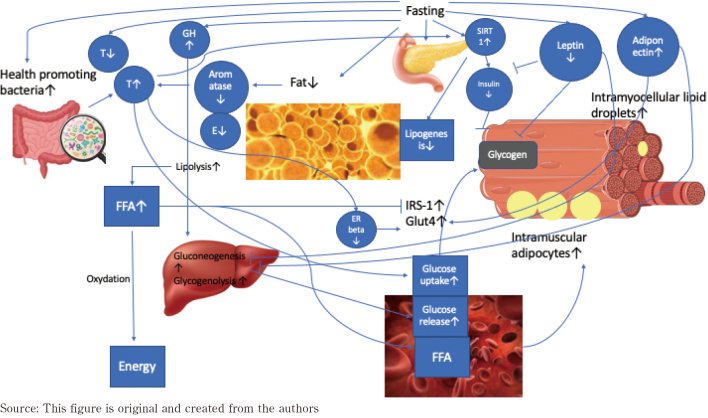
Hypothesized mechanism by which fasting affects lipid and glucose metabolism Fasting improves bacteria in the gut, leading to increased testosterone production, increased glucose uptake, and glycogen storage. Fasting also increases growth hormone release, increasing gluconeogenesis and glycogenolysis. Fasting decreases lipogenesis, increases lipolysis, leading to free fatty acid release and energy. Fasting decreases leptin and insulin release, while increasing adiponectin, leading to upregulated use of glucose. FFA: Free fatty acids, GH: Growth hormone, GLUT4: Glucose transporter type 4, E: Estradiol, ERbeta: Estrogen receptor beta, IRS-1: Insulin receptor substrate-1, SIRT1: Sirtuin 1 T: Testosterone

Even though acute periods of fasting might decrease serum testosterone, a mechanism potentially involved in the regulation of testosterone via fasting might be the reduction in body fat associated with limited food intake and the following decreases in aromatizing enzymes converting testosterone into estradiol. It is well known that adipose tissue is associated with increased levels of aromatase, the enzyme converting testosterone into estradiol. On the one hand, testosterone improves glucose uptake via GLUT 4 translocation, leading to improved glucose metabolism^57)^. Moreover, testosterone and androgens in general reduce the expression of estrogen receptor beta (ERbeta) activity which down-regulates GLUT 4 activity. Low testosterone levels increase ERbeta expression triggering the down-regulation of GLUT4, leading to impaired insulin resistance^58)^, while increased estradiol levels intensify the effects on ERbeta. The combination of decreased testosterone and increased estradiol amplifies the disordered glucose homeostasis and insulin resistance^58)^. Therefore, fasting can be beneficial for increasing testosterone levels via reduction of adipose tissue and the down-regulation of the aromatase enzyme, resulting in improved glucose control. Figure 1 summarizes the effects of fasting on lipid and glucose metabolism.

## Fasting and circadian rhythms

Time-restricted feeding seems to affect oscillations of the mRNA expression of circadian clock genes^59)^. In the attempt to deal with daily cycles of nutrient availability, our energy metabolism systems have shifted to cyclical mechanisms. This is the response from cell autonomous circadian rhythms and the feeding-fasting cycle. Cell autonomous circadian rhythms depend on negative feedback loops where bHLH-PAS transcription factors BMAL1, CLOCK, NPAS2 and ROR proteins activate and PER, CRY and REV-ERB inhibit transcription, leading to a 24 hours rhythmic transcription of target genes^60)^.

Feeding and fasting also regulate daily rhythms of major regulators of nutrient homeostasis such as AMPK, CREB and AKT. A strong feedback mechanism between circadian oscillator components and the feeding-fasting metabolic regulators activates coordinated oscillations at the transcript level and upregulates a large number of neuroendocrine, signaling and metabolic pathways. Disruption in circadian oscillators can trigger obesity and diabetes. Hence, daily rhythms of feeding and fasting might be crucial to maintain a healthy metabolism^60)^. Interestingly, time restricted fasting has also been shown to alternate the circadian rhythm of testosterone secretion^61)^. Moreover, it has been documented that food intake can acutely decrease testosterone levels, hence alternating the circadian rhythm^62)^. Regulators of nutrient homeostasis such as Akt signaling also regulate skeletal muscle mass through activation of mTOR, a pathway which is a target of androgen signaling. Testosterone decreases have been associated with impaired Akt phosphorylation, and the phosphorylation of Akt targets^63)^. From these findings, we can suggest a fine interplay between proteins and hormones affected by the circadian rhythms and fasting.

## Side effects of fasting

Besides the many health benefits of fasting, mild side effects can occur in some individuals, depending on their lifestyles and fasting style.

### Headaches

In fasting coffee drinkers, caffeine withdrawal can lead to headaches. Hypoglycemia can also trigger headache^64)^.

### Heartburn

Consuming large amounts of food after a period of fasting can lead to heartburn and gastroesophageal acid reflux, especially with fried and spicy foods^65)^.

### Constipation

Longer periods of fasting and lower fluid intake may reduce bowel movements, and lead to constipation in some individuals, especially if the diet if low in fibers^65)^.

### Dehydration

Liquid consumption can decrease while fasting, potentially leading to dehydration^66)^.

### Anemia

Fasting can lead to a reduction of hemoglobin, increasing the risk of anemia^67)^.

## Conclusion

A long time before the health benefits of fasting were identified, people practiced fasting for religious reasons. Nowadays, intermittent fasting has been deeply investigated and has become a health trend in our modern society. However, it is just in recent years that we partially understand the exact pathways involved in the health benefits triggered by fasting. The specific modalities maximizing the effects of fasting are also still not completely understood. The pathways involved in fasting-induced metabolic function shifts involve multiple factors such as sex hormones, lipid-released hormones, growth hormone, insulin, and the gut microbiome. Fasting activates lipolysis and the release of FFA into the bloodstream but also stored inside the muscle cells in the form of intramuscular adipocytes or intramyocellular lipid droplets. Insulin is downregulated and leads to a decrease in glycogen storages in the muscle cells. Even though the direct effects of fasting might lead to decreased testosterone levels, testosterone is also indirectly upregulated via downregulation of aromatase activity due to fat loss. Increased testosterone leads to the upregulation of IRS-1 and GLUT4, improving glucose metabolism. GH is upregulated in response to fasting, leading to increases in gluconeogenesis and glycogenolysis in the liver, leading to upregulated glucose release into the blood stream. GH also upregulates lipolysis and the following release of FFA. Leptin is downregulated and adiponectin is upregulated in response to fasting, leading to decreased hepatic gluconeogenesis and improved insulin sensitivity.

We can clearly observe positive effects of fasting on lipid and glucose metabolism. However, further research comparing several fasting modalities is necessary in order to understand which fasting period maximizes theses effects on metabolic functions. Direct comparisons among several fasting methods involving several variables such as duration, frequency, calories, and time of the day leading to numerous combinations represent a great potential for future research with regard to optimal fasting protocols. Also, long-term studies investigating the effects of fasting on adipose tissue reduction and serum testosterone and GH levels would greatly benefit the understanding of the specific mechanisms those hormones affect our well-being.

## Funding

No funding was received.

## Author contributions

JF designed and wrote the manuscript. TM reviewed and corrected the manuscript. SH designed and reviewed the manuscript.

## Conflicts of interest statement

The authors have no conflict of interest.
